# Autologous Stem Cell Transplantation in HIV-Positive and HIV-Negative Patients with Lymphoma: A Propensity Score-Matched Comparative Analysis

**DOI:** 10.3390/cancers18040584

**Published:** 2026-02-10

**Authors:** Alessandro Re, Margherita Oberti, Armando Stabile, Angelo Andreini, Chiara Cattaneo, Chiara Pagani, Salvatore Casari, Maria Antonia Forleo, Cristina Tecchio, Camillo Almici, Alessandra Tucci, Francesco Castelli, Giuseppe Rossi, Mauro Krampera

**Affiliations:** 1 UOC di Ematologia, ASST Spedali Civili di Brescia, 25123 Brescia, Italy; margherita.oberti@asst-spedalicivili.it (M.O.); chiara.cattaneo@asst-spedalicivili.it (C.C.); chiara.pagani@asst-spedalicivili.it (C.P.); alessandra.tucci@asst-spedalicivili.it (A.T.); giuseppe.rossi@asst-spedalicivili.it (G.R.); 2 UOC di Ematologia, Azienda Ospedaliera Universitaria Integrata Verona, 37122 Verona, Italy; angelo.andreini@univr.it (A.A.); cristina.tecchio@univr.it (C.T.); mauro.krampera@univr.it (M.K.); 3 IRCCS Ospedale San Raffaele, Vita-Salute, Università San Raffaele, 20132 Milano, Italy; armando.stabile88@gmail.com; 4 Division of Infectious Disease, ASST Spedali Civili di Brescia, 25123 Brescia, Italy; salvatore.casari@asst-mantova.it (S.C.); maria.forleo@unibs.it (M.A.F.); francesco.castelli@unibs.it (F.C.); 5 SC di Malattie Infettive, ASST Mantova, 46100 Mantova, Italy; 6 Immunoematologia e Medicina Trasfusionale, ASST Spedali Civili di Brescia, 25123 Brescia, Italy; camilloalmici@gmail.com; 7 Unit of Infectious Diseases, Department of Clinical and Experimental Sciences, Università degli Studi di Brescia, 25123 Brescia, Italy

**Keywords:** HIV, lymphoma, autologous stem cell transplantation

## Abstract

Background/Objectives: Treatment of HIV-associated lymphoma (HIV-Ly) with autologous stem cell transplantation (ASCT) has shown a surprisingly low relapse rate in several series. The aim of this study was to compare the clinical outcomes of HIV-Ly and lymphomas in the general population receiving ASCT. Methods: We compared two series of consecutive HIV-positive and HIV-negative patients, based on a 1:1 propensity score analysis, matching for age, sex, histology, disease status and prior therapies. Results: After propensity matching we identified a final population of 88 patients (44 HIV-positive vs. 44 HIV-negative). All HIV-positive patients received combination antiretroviral therapy (cART). With a median follow-up of 51 months, PFS was significantly higher in HIV-positive patients (4-year PFS 81% and 51%, in HIV-positive and HIV-negative patients, respectively, *p* = 0.027). Four-year OS was 81% for HIV-positive and 67% in HIV-negative patients (*p* = 0.15). The relapse rate was significantly higher in HIV-negative patients (36% vs. 23%) (*p* = 0.04). Conclusions: Our results clearly show that ASCT is an effective curative option for HIV-Ly, with better PFS and a lower relapse rate compared to patients without HIV. A favorable effect of ASCT on HIV infection and immune system recovery, potential off-target effects of cART or other yet unknown factors may account for this observation.

## Simple Summary

Treatment of HIV-associated lymphoma (HIV-Ly) with autologous stem cell transplantation (ASCT) has shown to be feasible and effective. In this study we compare the clinical outcomes of HIV-Ly and lymphomas of the general population receiving ASCT, analyzing two series of consecutive HIV-positive and HIV-negative patients, based on a 1:1 propensity score match analysis. Forty-four patients were identified in both groups. Progression-free survival (PFS), defined as the time from ASCT to relapse, progression or death for any cause, was significantly higher in HIV-positive patients (4-year PFS 81% and 51%, in HIV-positive and HIV-negative patients, respectively, *p* = 0.027). The 4-year OS was 81% in HIV-positive and 67% in HIV-negative patients (*p* = 0.15). The relapse rate was significantly higher in HIV-negative patients (36% vs. 23%) (*p* = 0.04). Our results increase the awareness of ASCT as an effective curative option for HIV-Ly and show for the first time better PFS and a low relapse rate after ASCT in patients with HIV compared to patients without, in a statistically reliable manner.

## 1. Introduction

The global incidence of HIV-associated lymphoma (HIV-Ly) decreased consistently after the widespread use of combination antiretroviral therapy (cART). Nevertheless Hodgkin (HL) and non-Hodgkin lymphoma (NHL) remain a major cause of morbidity and mortality in people living with HIV (PLWH) [1,2]. Treatment outcomes of HIV-Ly have improved in recent decades, due to effective cART and better supportive care, making standard therapeutic regimens established for HIV-negative patients also feasible in the HIV setting. Outcomes nowadays are similar in HIV-positive and HIV-negative patients with lymphoma when treated with the same standard therapies [3]. However, several population-based studies have highlighted a major health care disparity between the HIV-positive and negative population and studies of novel cancer therapeutics still usually exclude patients with HIV infection [4,5].

The use of high-dose therapy (HDT) with autologous stem cell transplantation (ASCT) has been shown to be feasible and highly effective in HIV-Ly for several years [6,7,8,9,10]. To date ASCT is offered to HIV-positive patients with lymphoma with the same indication as for HIV-negative patients, at least in developed countries, and concerns about potentially higher infection toxicity do not limit the use of this procedure [11]. A surprisingly low relapse rate has been reported in some series of PLWH who received ASCT for HIV-Ly [7,12,13,14,15]. Some of these studies have compared the results of monocentric or multicentric cohorts of HIV-positive subjects with historical series of HIV-negative patients undergoing ASCT or with controls identified among HIV-negative groups of patients according to various matching criteria. Although the HIV-positive patients usually had a higher clinical risk, a trend in favor of a lower relapse rate and longer progression-free survival (PFS) has been reported for PLWH, although statistically not significant [11,13,14,15].

In this study, we performed a comparison between two series of consecutive HIV-positive and negative patients with lymphoma receiving HDT and ASCT at two Italian centers, based on a 1:1 propensity score match (PSM) analysis, considering the main confounding variables. The aim was to directly compare, in a statistically reliable manner, the impact of ASCT, in terms of clinical outcome and toxicity, according to patients’ HIV status.

## 2. Material and Methods

### 2.1. Study Design and Population

We compared the clinical outcome of a case series of HIV-Ly that underwent ASCT and a cohort of comparable HIV-negative patients with lymphoma who received the same treatment, using a propensity score system to appropriately match the two groups.

The HIV-positive cohort comprised 66 consecutive patients receiving HDT and ASCT at the Hematology Department of Spedali Civili di Brescia, Brescia, Italy, from January 2001 to December 2019. As the comparison group we chose two consecutive series of HIV-negative patients who underwent ASCT at the Hematology Unit of Spedali Civili di Brescia, Brescia, Italy and at the Policlinico Borgo Roma, Verona, Italy, to increase the likelihood of finding adequately matched cases. Patients who underwent the first ASCT in a tandem program of autologous–autologous or autologous–allogeneic transplantation were excluded as patients who had prior ASCT or allogeneic transplantation. Two hundred and eighty-five HIV-negative patients between 2010 and 2017 were identified.

All patient data were collected from an anonymized database previously gathered with patients’ consent, for research and publication purposes. To adjust for confounders in baseline differences between the two groups, a balance was achieved using a 1:1 PSM analysis that led to a final population of 88 patients (44 HIV-positive and 44 HIV-negative). Matching criteria included age, sex, histology, disease status at the time of transplant and number of previous therapy regimens. Disease status at ASCT was classified according to the following three categories: first complete remission (CR); chemotherapy-sensitive disease (chemo-s), which includes partial response (PR) after first-line therapy, or chemotherapy-sensitive disease (at least PR) after salvage therapy; and chemotherapy-resistant disease (chemo-r), when less than PR was achieved with the latest therapy received. Disease response was defined by the Revised Response Criteria for Malignant Lymphoma [16]. All cases were diagnosed by expert pathologists, and a histological review was deemed unnecessary.

The primary objective of this study was to compare the PFS of the two groups of HIV-positive and negative patients. Secondary objectives included evaluation of overall survival (OS), relapse rates (RR), toxicity, mortality rates, and evaluation of the causes of death. Then, the primary endpoint was PFS and secondary endpoints were OS; RR; toxicity according to the Common Terminology Criteria for Adverse Events, version 5.0 [17]; pre- and post-infectious event rates; mortality rates; lymphoma-specific mortality (LSM); and other-cause mortality (OCM) rates.

### 2.2. Statistics and Definition of Outcomes

Statistical analyses comprised a few steps. First, medians and interquartile ranges (IQR) or frequencies and proportions were reported for continuous or categorical variables, respectively. A Mann–Whitney U test and a Chi-square test were applied to determine the statistical significance of differences in medians and proportions, respectively. Second, propensity score match analysis was performed to achieve a comparable cohort of patients affected and not affected by HIV, respectively. Propensity scores were computed for each patient using the 1:1 nearest-neighbor approach with a caliper width of 0.1, by modeling logistic regression with the dependent variable as the presence of HIV and accounting for the following confounders: age, sex (male, female), histology, disease status at ASCT (1st CR, chemo-s, chemo-r) and the number of prior lines of therapy (1, 2, ≥3). Third, survival analyses were performed by means of Kaplan–Meier curves, using a log-rank test to compare different groups. Fourth, Cox regression analysis was performed for univariable survival analyses. For univariate analysis of prognostic factors for survival, the following parameters were considered: age, sex, histology, disease status at transplant, and number of prior lines of therapy. Finally, cumulative incidence analyses were used to plot LSM and OCM rates. PFS was defined as the time from ASCT to relapse, progression or death for any cause. OS was measured from ASCT to death for any cause, or the last time the patient was known to be alive. LSM was defined as mortality due to active lymphoma, and OCM as mortality due to any cause other than disease relapse or progression. Neutrophil engraftment was defined as the first day with an absolute neutrophil count greater than 500/μL and platelet engraftment as a self-supporting platelet count greater than 20,000/μL.

Grade > 2 toxicities were considered, according to the Common Terminology Criteria for Adverse Events, version 5.0. Infectious events were classified by post-ASCT time frames: pre-engraftment period (PRE-EP) refers to the period between HDT and hematological engraftment and post-engraftment period (POST-EP) refers to the time frame after the hematological engraftment and the first two years from ASCT. All analyses were performed using the R software v.3.6.3. All tests were two-sided with a significance level set at *p* < 0.05.

## 3. Results

### 3.1. Patients’ Characteristics

**Figure 1:**
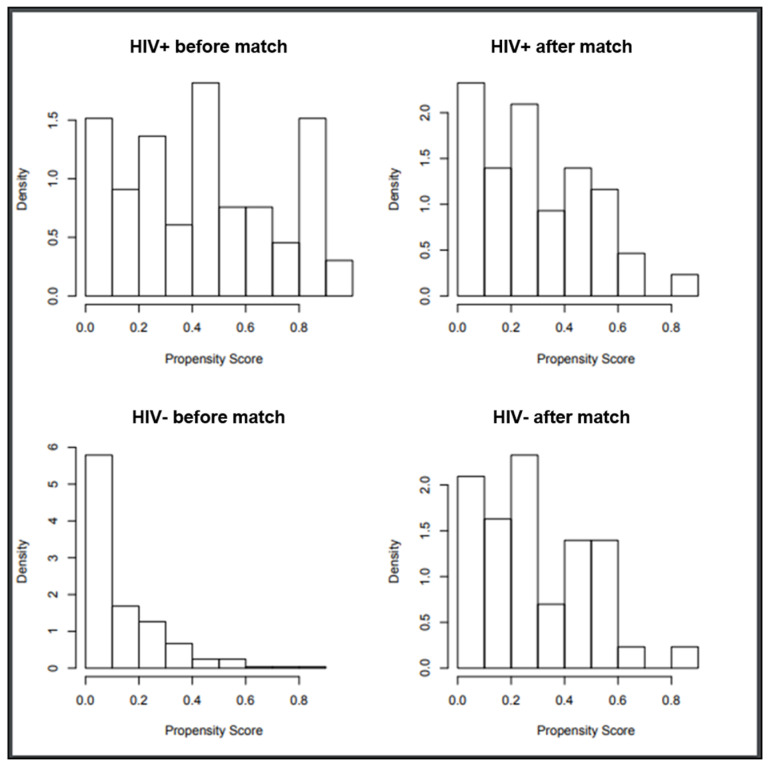
Propensity score distribution of the 2 cohorts, HIV-positive and negative patients, before and after propensity score-based nearest-neighbor 1:1 matching.

Following propensity score-based nearest-neighbor 1:1 matching, we identified 44 HIV-positive patients (from the initial 66) and 44 HIV-negative patients (from the initial 285) (Figure 1). Baseline patients’ characteristics are shown in Table 1. Half of the patients had a Diffuse Large B-cell Lymphoma, the great majority had a chemo-s disease and 50% had received more than two lines of previous therapy. Within the HIV-positive population, the median CD4+ T-cells before ASCT was 219/µL (range 60–720) and only one patient (2%) had detectable HIV viremia. All patients were on cART at the time of ASCT. Only two patients discontinued cART for at least 1 week after ASCT due to oral mucositis and hepatic toxicity, respectively. Co-infection with hepatitis B and hepatitis C virus was present in 1 (2%) and 12 (27%) HIV-positive patients and in 1 (2%) and 5 (11%) HIV-negative patients.

### 3.2. Clinical Outcomes

**Figure 2:**
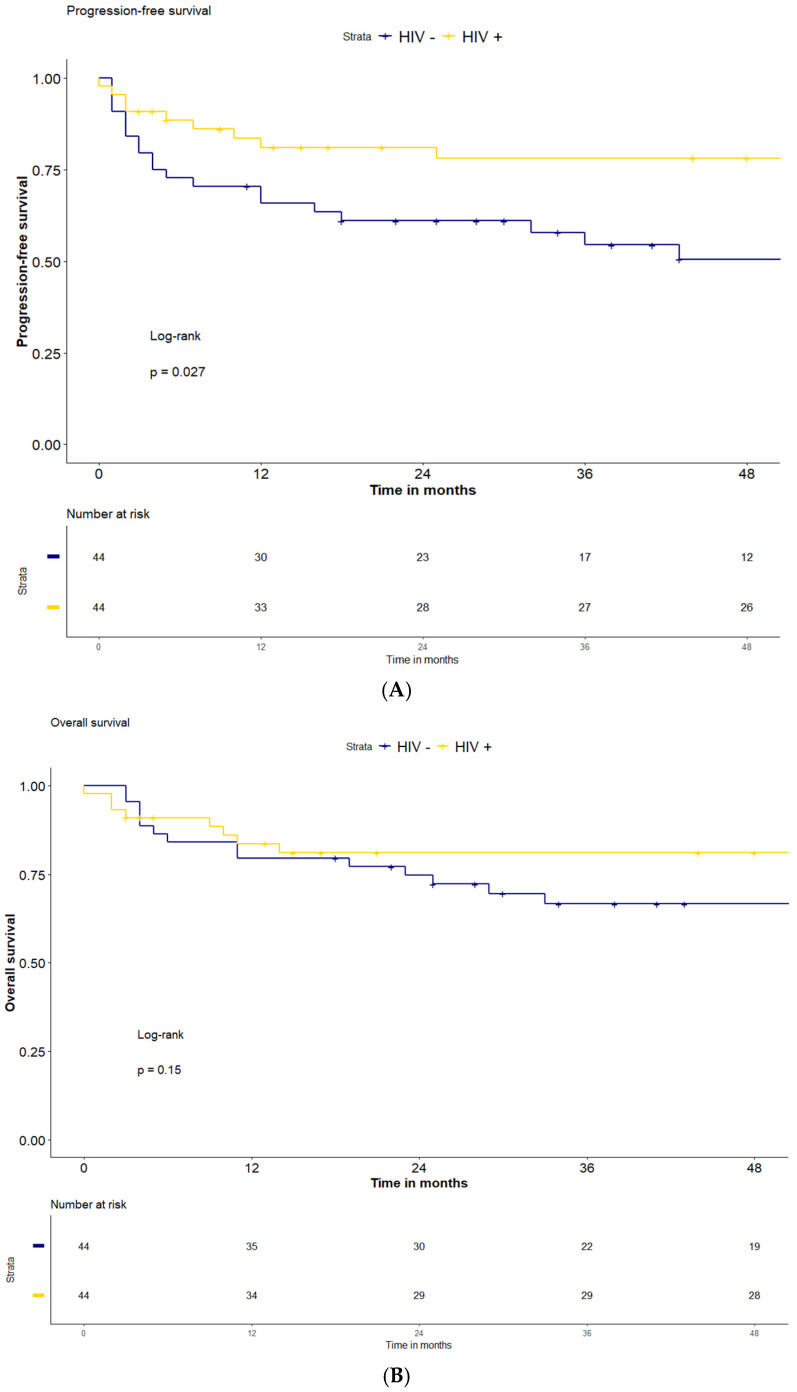
Survival according to HIV infection status. PFS (**A**) and OS (**B**) for HIV-positive and negative patients with lymphoma receiving ASCT.

Overall, after a median follow-up of 51 months (IQR: 15–80), the 2-year and 4-year PFS were 71% and 65%, and the 2-year and 4-year OS were 78% and 74% for the entire series of 88 patients. PFS in the HIV-positive group was significantly higher compared to the HIV-negative population (2-year and 4-year PFS 81% and 78% vs. 61% and 51%, respectively, *p* = 0.027) (Figure 2). The 2-year OS and 4-year OS were both 81% for the HIV-positive patients and 75% and 67% for the HIV-negative patients (*p* = 0.15). The median follow-up for the two groups was 51 months (IQR: 8–87) for HIV-positive and 43 (IQR 24–73) for the HIV-negative population.

Overall, 30 patients (34%) had lymphoma relapse after ASCT. The relapse rate was significantly higher in HIV-negative (20 patients, 45%) compared to HIV-positive patients (10 patients, 23%) (*p* = 0.04). The median time to relapse was 57 months (IQR 12–89) for HIV-positive versus 27 months (IQR 5–53) for HIV-negative patients (*p* = 0.03). Twenty-six cases of death were recorded. Ten patients (23%) in the HIV-positive group died: seven from lymphoma, two from infection during CR (one had stopped cART by his own decision several months after ASCT), and one due to suicide. In the HIV-negative group, 16 patients (36%) died: 11 from lymphoma, four from infection (one patient in CR due to pneumonia, 11 months after ASCT, and three while receiving further therapies for lymphoma relapse), and one from a second tumor (pancreatic adenocarcinoma).

**Figure 3:**
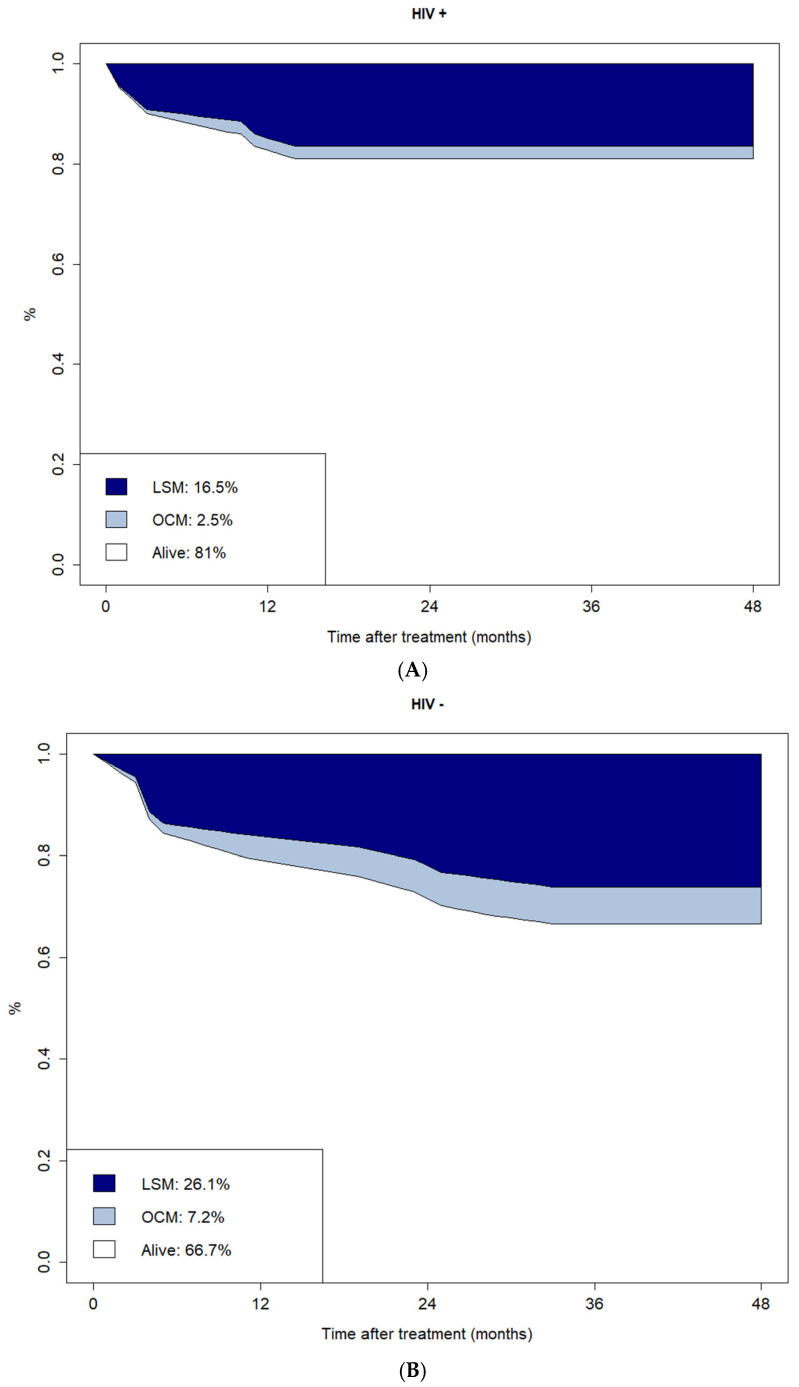
Cumulative incidence plot of Lymphoma-Specific Mortality (LSM) and Other-Cause Mortality (OCM) according to HIV infection status. LSM and OCM at 4 years for HIV-positive and negative patients with lymphoma, receiving ASCT, are reported in Figure (**A**) and Figure (**B**) respectively.

The CI plots of LSM and OCM are reported in Figure 3. The CIs of LSM and OCM at four years for HIV-positive versus HIV-negative patients were 16.5% vs. 26.1% and 2.5% vs. 7.2%, respectively.

### 3.3. Engraftment, Toxicity, and Infections

In all patients, peripheral hematopoietic stem cells were used for transplantation. The median number of CD34+ cells infused was 6.3 (IQR 5.0–7.3) × 10^6^/kg in the HIV-positive and 5.75 (IQR 4.9–7.9) × 10^6^/kg in the HIV-negative patients (*p* = 0.72). Neutrophil and platelet engraftment was achieved in all patients, except for one HIV-positive patient with chemo-r disease who died 10 days after ASCT because of sepsis. The median time to neutrophil and platelet engraftment was 10 days and 13 days for both HIV-positive and negative patients.

As infection prophylaxis HIV-negative patients received acyclovir and trimethoprim sulfamethoxazole until 3 months after ASCT. HIV-positive patients received acyclovir and fluconazole for 3 months after ASCT, and trimethoprim sulfamethoxazole for at least 6 months (or until CD4 count > 200/mcl). Levofloxacin was used in both groups during the neutropenic period before engraftment.

Eighteen (41%) HIV-positive patients had a total of 20 grade 3–4 treatment-related toxicity events (10 oral mucositis, 6 gastrointestinal toxicity/diarrhea, 3 enteritis, 1 seizure), while in the HIV-negative group, 16 grade 3–4 toxicity events were seen in 15 (34%) patients (14 oral mucositis, 2 enteritis) (*p* = 0.6). Nineteen patients (43%) in the HIV-positive group had an episode of fever of unknown origin (FUO) versus 26 (59%) in the HIV-negative group (*p* = 0.2). During the PRE-EP, there was a similar number of infectious events between the two groups (15 events in 14 patients and 11 events in 9 patients within the HIV-positive and negative group, respectively, *p* = 0.48). A documented bacterial infection was seen in 9 HIV-positive patients (20%) (including 3 g-negative and 4 g-positive episodes of sepsis), and in 7 HIV-negative patients (16%) (including 3 g-negative and 1 g-positive episodes of sepsis). A pneumonitis was recorded in one HIV-positive and two HIV-negative patients. Two cases (4%) of asymptomatic cytomegalovirus (CMV) reactivation, one (2%) herpes zoster (HZV) infection, and one (2%) human herpes virus-6 (HHV-6) infection were seen in the HIV-positive group while only one (2%) HHV-6 infection was observed in the HIV-negative group. One *C.parapsilosis* sepsis was observed in one HIV-negative patient and one HIV-positive patient had a probable invasive pulmonary aspergillosis. During the POST-EP there was a significant majority of infections or febrile events in the HIV-positive population compared to the HIV-negative group (34 vs. 17, respectively, *p* = 0.02). A total of 29 documented late infections occurred in 23 HIV-positive patients, including 5 bacterial infections, 20 viral infections (8 CMV, 11 HZV, 1 Rhinovirus pneumonia), and 4 cases of pneumonia. In the HIV negative-population, 12 patients had 15 documented infection events: two bacterial infections, 10 viral (7 CMV and 3 HZV), and 3 cases of pneumonia.

### 3.4. Predictive Factors for Relapse and Mortality

Considering the whole series of 88 patients, at univariable Cox regression analysis, two treatment lines prior to ASCT (HR: 0.07; *p* = 0.01) and chemo-refractory disease at ASCT (HR: 32; *p* < 0.01) significantly correlated with lower PFS. HIV infection resulted in being a protective factor for PFS (HR: 0.43; *p* = 0.03). Age (HR: 1.04; *p* = 0.03), NHL T histology (HR: 3.1; *p* = 0.05) and chemo-refractory disease at ASCT (HR: 6.6; 95% CI: 1.6–27; *p* = 0.01) were significantly correlated with a higher risk of death.

## 4. Conclusions

Thanks to the widespread use of cART, and the improvement in supportive care, standard-dose chemotherapy and HDT with ASCT are nowadays offered to HIV-positive patients with lymphoma with the same indications as for the HIV-negative general population.

However, access to target therapies and modern immunotherapies is still limited to PLWH, due to socio-economic factors and inadequate research to support safety and efficacy in this population. In the present study, we compared the outcomes of ASCT among two series of lymphoma patients affected or not affected by HIV infection using a 1:1 propensity score matching to reduce the bias of the retrospective comparison. Unpredictable satisfactory outcomes were previously reported for HIV-positive patients, comparable to HIV-negative subjects, with a surprising trend toward less relapses in the HIV-positive population [11,13,14,15].

The main result of our study was the significantly lower relapse rate in the HIV-positive group, which resulted in statistically significant higher PFS in PLWH compared to HIV-negative patients. This has also resulted in a not statistically significant trend toward improved OS in HIV-positive patients. PFS and OS of HIV-positive patients in the present study are in line with previous reports [9,18].

The fascinating finding of better PFS in patients with HIV infection compared to the general population is difficult to interpret. A beneficial effect of ASCT on the underlying HIV infection and on the immune status of patients with HIV may be hypothesized. Highly myelotoxic chemotherapy could deplete the reservoir that harbors HIV and facilitates immune recovery, and the restoration of an effective immune system might be relatively more beneficial in lymphomas developing in the context of failing immune response than in immunocompetent patients [19]. In hematologic malignancies, there are several examples of immunodeficiency-driven diseases that benefit from immune reconstitution, by simply reducing or modifying immune suppression, and several cases of lymphoma regression with treatment of HIV alone have been reported [20,21,22]. Moreover, a favorable long-term effect of cART may also play a role in the reduction in the relapse risk in HIV-Ly. Antiviral drugs included in cART regimens have been reported to exert a direct antineoplastic action [23,24,25]. Moreover, several studies have demonstrated the efficacy of the antiretroviral zidovudine, used in combination with interferon-α, against some adult T-cell leukemia subtypes, a disease etiologically associated with the human T-cell leukemia retrovirus type I. It is uncertain whether these drugs function via antiviral activity in cells in the tumor or microenvironment or through mechanisms other than antiviral activity [26]. Even if antiviral drugs have not been approved as antiproliferative agents, promising in vitro studies have demonstrated that nucleoside/nucleotide analog, reverse transcriptase inhibitors or integrase inhibitors can modulate cell growth and differentiation across various cancer types [27,28,29]. Investigations are ongoing to identify agents with the ability to inhibit key enzymes that play a crucial role in DNA metabolism and their possible application as antiretroviral and antitumoral agents [30].

In our study, we did not find significant differences both in acute toxicity and early infections after ASCT between the two groups of HIV-positive and negative patients. Instead, infections occurring later after engraftment were significantly higher in PLWH, and were mainly of viral origin. An increased rate of infections was also reported by the Spanish group [11], without a detrimental effect on survival. A previous study found a comparable immunologic recovery after ASCT between HIV-positive and negative patients, while other studies reported a delayed recovery in PLWH irrespective of efficient cART [11,19]. It is also known that chronic inflammation, immune cell metabolic dysregulation, and cellular dysfunction persist in PLWH even in the presence of effective cART and suppressed HIV viremia [31]. Our experience underlines the need for optimal supportive measures and careful follow-up in HIV-positive patients. Anyway, in our study not only LSM but also OCM showed a trend in favor of PLWH.

In conclusion, our results increase the awareness of ASCT as an effective curative option for patients with HIV-Ly and chemo-sensitive disease. ASCT indeed appeared to be highly effective in our study, and we reported for the first time a significantly lower relapse rate in HIV-positive patients compared to the general HIV-negative population.

We are aware that future clinical studies in the HIV setting should focus on novel molecules and immunological approaches while considering the effects on the underlying HIV disease, and some preliminary experiences paved the way in this direction [32,33,34]. While waiting for these new therapeutic approaches, rapidly developing in the HIV-negative population [35], to be demonstrated as safe and effective in the setting of HIV, ASCT remains a highly effective treatment strategy for HIV-Ly. A collaboration between all the involved professional figures, particularly infectious disease specialists and hemato-oncologists, represents the key to the correct management of these patients to guarantee the best achievable oncological outcome.

## Figures and Tables

**Table 1:** Descriptive characteristics of 88 patients with lymphoma (44 HIV-positive and 44 HIV-negative) receiving ASCT, after propensity score matching.

Variables	Overall n = 88	HIV − (n = 44)	HIV + (n = 44)	*p*-Value
Age at ASCT, yrs				0.7
Median	47	46	47	
IQR	36–54	35–57	38–54	
Sex, n (%)				0.8
M	78 (89)	38 (86)	40 (91)	
F	10 (11)	6 (14)	4 (9)	
Histology, n (%)				
HL	24 (27)	13 (30)	11 (25)	0.9
DLBCL/PBL	44 (50)	20 (46)	24 (54)	
Burkitt/Burkitt-like Lymphoma	4 (5)	2 (4.5)	2 (4.5)	
PTCL	11 (13)	6 (14)	5 (11)	
FL	3 (3.4)	2 (4.5)	1 (2.3)	
PMBCL	2 (2.3)	1 (2.3)	1 (2.3)	
Status at ASCT, n (%)				
1st CR	13 (15)	3 (7)	10 (23)	0.1
Chemo-s	68 (77)	37 (84)	31 (71)	
Chemo-r	7 (8)	4 (9)	3 (7)	
Number of prior therapies, n (%)				
1	22 (25)	12 (27)	10 (23)	0.6
2	22 (25)	9 (21)	13 (30)	
≥3	44 (50)	23 (52)	21 (48)	

IQR: interquartile range. ASCT: autologous stem cell transplantation. HL: Hodgkin Lymphoma; DLBCL/PBL: Diffuse Large B-cell Lymphoma/Plasmablastic Lymphoma (PBL was merged with DLBCL as it was identified only in the HIV-positive group); PTCL: Peripheral T-cell Lymphoma; FL: Follicular Lymphoma; PMBCL: Primary Mediastinal B-cell Lymphoma; Chemo-s: chemo-sensitive disease; Chemo-r: chemo-resistant disease.

## Data Availability

The original contributions presented in the study are included in the article, further inquiries can be directed to the corresponding author.

## References

[1] Robbins H.A., Pfeiffer R.M., Shiels M.S., Li J., Hall I., Engels E.A. (2015). Excess cancers among HIV-infected people in the United States. J. Natl. Cancer. Inst..

[2] Kimani S.M., Painschab M.S., Horner M.J., Muchengeti M., Fedoriw J., Shiels M.S., Gopal S. (2020). Epidemiology of haematological malignancies in people living with HIV. Lancet HIV.

[3] Re A., Cattaneo C., Montoto S. (2020). Treatment management of haematological malignancies in people living with HIV. Lancet Haematol..

[4] Coghill A., Suneja G. (2017). Cancer care disparities in people with HIV in the United States. Curr. Opin. HIV AIDS.

[5] Uldrick T.S., Ison G., Rudelk M.A., Noy A., Schwartz K., Bruinooge S., Schenkel C., Miller B., Dunleavy K., Wang J. (2017). Modernizing Clinical Trial Eligibility Criteria: Reccomendations of the American Society of Clinical Oncology-Friends of Cancer Research HIV Working Group. J. Clin. Oncol..

[6] Gabarre J., Azar N., Autran B., Katlama C., Leblond V. (2000). High-dose therapy and autologous haematopoietic stem-cell transplantation for HIV-1-associated lymphoma. Lancet.

[7] Re A., Cattaneo C., Michieli M., Casari S., Spina M., Rupolo M., Allione B., Nosari A., Schiantarelli C., Vigano M. (2003). High-dose therapy and autologous peripheral-blood stem-cell transplantation as salvage treatment for HIV-associated lymphoma in patients receiving highly active antiretroviral therapy. J. Clin. Oncol..

[8] Krishnan A., Molina A., Zaia J., Nademanee A., Kogut N., Rosenthal J., Woo D., Forman S.J. (2001). Autologous stem cell transplantation for HIV associated lymphoma. Blood.

[9] Re A., Michieli M., Casari S., Allione B., Cattaneo C., Rupolo M., Spina M., Manuele R., Vaccher E., Mazzucato M. (2009). High-dose therapy and autologous peripheral blood stem cell transplantation as salvage treatment for AIDS-related lymphoma: Long-term results of the Italian Cooperative Group on AIDS and Tumors (GICAT) study with analysis of prognostic factors. Blood.

[10] Balsalobre P., Díez-Martín J.L., Re A., Michieli M., Ribera J.M., Canals C., Rosselet A., Conde E., Varela R., Cwynarski K. (2009). Autologous stem-cell transplantation in patients with HIV-related lymphoma. J. Clin. Oncol..

[11] Bastos-Oreiro M., Balsalobre P., Miralles P., Berenguer J., Dorado N., Bailen R., Obreoscoa G., Anguita J., Serrano D., Díez-Martín J.L. (2020). Autologous stem cell transplantation for lymphoma in HIV+ patients: Higher rate of infections compared with non-HIV lymphoma. Bone Marrow Transplant..

[12] Re A., Gini G., Rupolo M., Levis A., Bandera A., Liberati A.M., Tozzi P., Cattaneo C., Casari C., Skert C. (2018). Early consolidation with high-dose therapy and autologous stem cell transplantation is a feasible and effective treatment option in HIV-associated non-Hodgkin lymphoma at high risk. Bone Marrow Transplant..

[13] Díez-Martín J.L., Balsalobre P., Re A., Michieli M., Ribera J.M., Canals C., Conde E., Rosselet A., Gabriel I., Varela R. (2009). Comparable survival between HIV+ and HIV− non-Hodgkin and Hodgkin lymphoma patients undergoing autologous peripheral blood stem cell transplantation. Blood.

[14] Krishnan A., Palmer J.M., Zaia J.A., Tsai N.C., Alvarnas J., Forman S.J. (2010). HIV status does not affect the outcome of autologous stem cell transplantation (ASCT) for non-hodgkin lymphoma (NHL). Biol. Blood Marrow Transplant..

[15] Alvarnas J.C., Le Rademacher J., Wang Y., Little R.F., Akpek G. (2016). Autologous hematopoietic cell transplantation for HIV-related lymphoma: Results of the BMT CTN 0803/AMC 071 trial. Blood.

[16] Cheson B.D., Fisher R.I., Barrington S.F., Cavalli F., Schwartz L.H., Zucca E., Lister T.A. (2014). Recommendations for initial evaluation, staging, and response assessment of Hodgkin and non-Hodgkin lymphoma: The Lugano classification. J. Clin. Oncol..

[17] U.S. Department of Health and Human Services, National Institutes of Health, National Cancer Institute (2017). Common Terminology Criteria for Adverse Events (CTCAE).

[18] Hubel K., Re A., Boumendil A., Finel H., Hentrich M., Robinson S., Wyen C., Michieli M., Kanfer E., Diez-Martin J.L. (2019). Autologous stem cell transplantation for HIV-associated lymphoma in the antiretroviral and rituximab era: A retrospective study by the EBMT Lymphoma Working Party. Bone Marrow Transplant..

[19] Bertoli D., Re A., Chiarini M., Sottini A., Serana F., Giustini V., Roccaro A.M., Cattaneo C., Caimi L., Rossi G. (2016). B- and T-lymphocyte number and function in HIV^+^/HIV^−^ lymphoma patients treated with high-dose chemotherapy and autologous bone marrow transplantation. Sci. Rep..

[20] Lee M., Abousaud A., Harkins A., Marin E., Balasubramani D., Churnetski M.C., Peker D., Singh A., Koff J.L. (2023). Important Considerations in the Diagnosis and Management of Post-transplant Lymphoproliferative Disorder. Curr. Oncol. Rep..

[21] Lim D.H., Rhee J.Y., Park K.W. (2017). Stage IV advanced diffuse large B-cell lymphoma in human immunodeficiency virus infection with achieving cure by using highly active antiretroviral therapy alone: A case report. Int. J. STD AIDS.

[22] Atallah-Yunes S.A., Murphy D., Abdelmalak R., Mantle L., Ali S.S. (2019). Plasmablastic lymphoma achieving sustained remission with antiretroviral therapy alone. Eur. J. Haematol..

[23] Monini P., Sgadari C., Toschi E., Barillari G., Ensoli B. (2004). Antitumour effects of antiretroviral therapy. Nat. Rev. Cancer.

[24] Maksimovic-Ivanic D., Fagone P., McCubrey J., Bendtzen K., Mijatovic S., Nicoletti F. (2017). HIV-protease inhibitors for the treatment of cancer: Repositioning HIV protease inhibitors while developing more potent NO-hybridized derivatives?. Int. J. Cancer.

[25] Chow W.A., Jiang C., Guan M. (2009). Anti-HIV drugs for cancer therapeutics: Back to the future?. Lancet Oncol..

[26] Cook L.B., Fuji S., Hermine O., Bazarbachi A., Ramos J.C., Ratner L., Horwitz S., Fields P., Tanase A., Bumbea H. (2019). Revised Adult T-Cell Leukemia-Lymphoma International Consensus Meeting Report. J. Clin. Oncol..

[27] García-Trejo J.J., Ortega R., Zarco-Zavala M. (2021). Putative Repurposing of Lamivudine, a Nucleoside/Nucleotide Analogue and Antiretroviral to Improve the Outcome of Cancer and COVID-19 Patients. Front. Oncol..

[28] Akcora-Yildiz D., Gonulkirmaz N., Ozkan T., Beksac M., Sunguroglu A. (2023). HIV-1 integrase inhibitor raltegravir promotes DNA damage-induced apoptosis in multiple myeloma. Chem. Biol. Drug. Des..

[29] Şekeroğlu Z.A., Şekeroğlu V., Küçük N. (2021). Effects of Reverse Transcriptase Inhibitors on Proliferation, Apoptosis, and Migration in Breast Carcinoma Cells. Int. J. Toxicol..

[30] Garro H.A., Pungitore C.R. (2015). Coumarins as potential inhibitors of DNA polymerases and reverse transriptases. Searching new antiretroviral and antitumoral drugs. Curr. Drug Discov. Technol..

[31] Mu W., Patankar V., Kitchen S., Zhen A. (2024). Review Examining Chronic Inflammation, Immune Metabolism, and T Cell Dysfunction in HIV Infection. Viruses.

[32] Rubinstein P.G., Moore P.C., Bimali M., Lee J.Y., Rudek M.A., Chadburn A., Ratner L., Henry D.H., Cesarman E., DeMarco C.E. (2023). AIDS Malignancy Consortium; Lymphoma Study Association. Brentuximab vedotin with AVD for stage II-IV HIV-related Hodgkin lymphoma (AMC 085): Phase 2 results from an open-label, single arm, multicentre phase 1/2 trial. Lancet Haematol..

[33] El Zarif T., Nassar A.H., Adib E., Fitzgerald B.G., Huang J., Mouhieddine T.H., Rubinstein P.G., Nonato T., McKay R.R., Li M. (2023). Safety and Activity of Immune Checkpoint Inhibitors in People Living with HIV and Cancer: A Real-World Report From the Cancer Therapy Using Checkpoint Inhibitors in People Living with HIV-International (CATCH-IT) Consortium. J. Clin. Oncol..

[34] Hattenhauer T., Mispelbaum R., Hentrich M., Boesecke C., Monin M.B. (2023). Enabling CAR T-cell therapies for HIV-positive lymphoma patients—A call for action. HIV Med..

[35] D’Alò F., Bellesi S., Maiolo E., Alma E., Bellisario F., Malafronte R., Viscovo M., Campana F., Hohaus S. (2024). Novel targets and advanced therapies in Diffuse Large B Cell Lymphomas. Cancers.

